# Analysis of *IL12B* Gene Variants in Inflammatory Bowel Disease

**DOI:** 10.1371/journal.pone.0034349

**Published:** 2012-03-30

**Authors:** Jürgen Glas, Julia Seiderer, Johanna Wagner, Torsten Olszak, Christoph Fries, Cornelia Tillack, Matthias Friedrich, Florian Beigel, Johannes Stallhofer, Christian Steib, Martin Wetzke, Burkhard Göke, Thomas Ochsenkühn, Julia Diegelmann, Darina Czamara, Stephan Brand

**Affiliations:** 1 Department of Medicine II - Grosshadern, Ludwig-Maximilians-University (LMU), Munich, Germany; 2 Department of Preventive Dentistry and Periodontology, LMU, Munich, Germany; 3 Department of Human Genetics, Rheinisch-Westfälische Technische Hochschule (RWTH), Aachen, Germany; 4 Division of Gastroenterology, Brigham & Women's Hospital, Harvard Medical School, Boston, United States of America; 5 Department of Pediatrics, Hannover Medical School, Hannover, Germany; 6 Max-Planck-Institute of Psychiatry, Munich, Germany; Catholic University Medical School, Italy

## Abstract

**Background:**

*IL12B* encodes the p40 subunit of IL-12, which is also part of IL-23. Recent genome-wide association studies identified *IL12B* and *IL23R* as susceptibility genes for inflammatory bowel disease (IBD). However, the phenotypic effects and potential gene-gene interactions of *IL12B* variants are largely unknown.

**Methodology/Principal Findings:**

We analyzed *IL12B* gene variants regarding association with Crohn's disease (CD) and ulcerative colitis (UC). Genomic DNA from 2196 individuals including 913 CD patients, 318 UC patients and 965 healthy, unrelated controls was analyzed for four SNPs in the *IL12B* gene region (rs3212227, rs17860508, rs10045431, rs6887695). Our analysis revealed an association of the *IL12B* SNP rs6887695 with susceptibility to IBD (p = 0.035; OR 1.15 [95% CI 1.01–1.31] including a trend for rs6887695 for association with CD (OR 1.41; [0.99–1.31], p = 0.066) and UC (OR 1.18 [0.97–1.43], p = 0.092). CD patients, who were homozygous C/C carriers of this SNP, had significantly more often non-stricturing, non-penetrating disease than carriers of the G allele (p = 6.8×10^−5^; OR = 2.84, 95% CI 1.66–4.84), while C/C homozygous UC patients had less often extensive colitis than G allele carriers (p = 0.029; OR = 0.36, 95% CI 0.14–0.92). *In silico* analysis predicted stronger binding of the minor C allele of rs6887695 to the transcription factor RORα which is involved in Th17 differentiation. Differences regarding the binding to the major and minor allele sequence of rs6887695 were also predicted for the transcription factors HSF1, HSF2, MZF1 and Oct-1. Epistasis analysis revealed weak epistasis of the *IL12B* SNP rs6887695 with several SNPs (rs11889341, rs7574865, rs7568275, rs8179673, rs10181656, rs7582694) in the *STAT4* gene which encodes the major IL-12 downstream transcription factor STAT4 (p<0.05) but there was no epistasis between *IL23R* and *IL12B* variants.

**Conclusions/Significance:**

The *IL12B* SNP rs6887695 modulates the susceptibility and the phenotype of IBD, although the effect on IBD susceptibilty is less pronounced than that of *IL23R* gene variants.

## Introduction

The identification of the IL-23/Th17 pathway as a key regulator of intestinal immune homeostasis and proinflammatory responses in defense to microbial infection has elucidated new potential therapeutic targets in inflammatory bowel diseases (IBD) [1], [2], [3], [4]. Genome-wide association studies (GWAS) and large cohort studies demonstrated that *IL23R* [3], [5], [6], [7] and additional genes involved in Th17 differentiation (e.g., *IL12B*, *JAK2*, *TYK2*, *STAT3*, *CCR6*, *IL2/IL21* and *TNFSF15*) are associated with the susceptibility to Crohn's disease (CD) and partly also to ulcerative colitis (UC) [5], [8], [9], [10], [11]. Moreover, a pathway analysis using data from the Wellcome Trust Case Control Consortium (WTCCC) uncovered significant associations of CD and IL-12/IL-23 pathway components, harbouring 20 genes such as *IL12B*, *JAK2*, *STAT3* and *CCR6* [12]. Functionally, the proinflammatory cytokines IL-12 and IL-23 play critical and unique roles in bridging the innate and adaptive immune systems in IBD [4], [13] and are produced primarily by activated dendritic cells (DCs) and macrophages in response to microbial stimulation. IL-12 promotes the differentiation of naive CD4+ T cells into mature interferon-γ (IFN-γ)-producing Th1 effector cells and is a potent stimulus of natural killer and CD8+ T cells [14], [15]. In contrast, IL-23, a heterodimeric cytokine composed of a p19 subunit and a p40 subunit of which the latter is shared with IL-12, is required for the generation of memory T cells and drives differentiation of Th17 cells [16], [17]. Th17 cytokines such as IL-17A, IL-17F, IL-21, IL-22, IL-26 and the Th17 chemokine CCL20 have been particularly implicated as key cytokines involved in the intestinal inflammation of CD [1], [18], [19], [20], [21], [22], [23].

On the genetic level, *IL12B* encodes the IL-12 p40 subunit shared by IL-12 and IL-23 cytokines while *IL23R* encodes one of the two subunits of the IL-23 receptor [24]. Antibody therapy directed against the IL-12/IL-23 p40 subunit demonstrated some clinical efficacy in CD patients [25]. Since the first identification of *IL23R* as susceptibility gene in IBD by Duerr *et al.* [3], various GWAS and cohort studies have confirmed *IL23R* not only as a major susceptibility gene in IBD but also in the pathogenesis of other autoimmune diseases such as psoriasis [26], [27] and ankylosing spondylitis [28], implicating common proinflammatory pathways. Genetic variants in the *IL12B* region have also been associated with the susceptibility to psoriasis, ankylosing spondylitis, and infectious diseases such as leprosy and tuberculosis [29], [30], [31], [32], [33]. In contrast, data on *IL12B* variants and their role in asthma [34], [35], rheumatoid arthritis [36] or multiple sclerosis [37] remain controversial. While recent GWAS meta-analyses by Barrett *et al.* [5], Franke *et al.* [9], and Anderson *et al.* [10] established *IL12B* as IBD susceptibility gene, smaller studies showed inconsistent results [38], [39]. Cohort studies in the Spanish [38] and Japanese [39] population demonstrated an association of *IL12B* SNPs with the susceptibility to IBD; however with different results, reporting associations with CD susceptibility in the Japanese cohort (rs6887695) [39] and to UC (rs6887695) but not to CD susceptibility in the Spanish cohort [38]. Moreover, the phenotypic effects of *IL12B* and potential gene-gene interactions contributing to IBD susceptibility are largely unknown.

Since the exact role of *IL12B* for IBD susceptibility in the German population and IBD phenotype behaviour remains unclear, we aimed to perform a detailed genotype-phenotype analysis in a large IBD cohort and an analysis for potential epistatic interactions with other gene variants involved in IL-12 and IL-23 signaling and implicated in IBD susceptibility such as *IL23R* and *STAT4* [3], [40] which encodes the major IL-12-induced downstream transcription factor STAT4.

## Methods

### Ethics statement

The study was approved by the Ethics committee of the Medical Faculty of Ludwig-Maximilians-University Munich and written, informed consent was obtained from all patients prior to the study. Study protocols adhered to the ethical principles for medical research involving human subjects of the Helsinki Declaration (as detailed under: http://www.wma.net/en/30publications/10policies/b3/index.html).

### Study population and IBD phenotype assessment

Overall, the study population (n = 2196) consisted of 1231 IBD patients including 913 patients with CD, 318 patients with UC, and 965 healthy, unrelated controls, all of Caucasian origin. The demographic and clinical data (behaviour and location of IBD, disease-related complications, immunosuppressive therapy and history of previous surgeries) of the patients were recorded by analysis of patient charts and a detailed questionnaire including an interview at time of enrolment. The diagnosis of CD or UC was determined according to established guidelines based on endoscopic, radiological, and histopathological criteria. Patients with CD were assessed following the Montreal classification based on the age at diagnosis (A), location (L), and behaviour (B) of the disease [41]. In patients with UC, anatomic location was also based on the Montreal classification using the criteria ulcerative proctitis (E1), left-sided UC (distal UC; E2), and extensive UC (pancolitis; E3). Patients with indeterminate colitis were excluded from the study. The demographic characteristics of the IBD study population were collected blind to the results of the genotype analyses and are summarized in Table 1.

**Table 1:** Demographic characteristics of the IBD study population.

	Crohn's disease*n = 913*	Ulcerative colitis*n = 318*	Controls*n = 965*
**Gender**			
Male (%)	48.9	52.2	63.5
Female (%)	51.1	47.8	36.5
**Age (yrs)**			
Mean ± SD	40.9±13.2	44.2±14.8	46.0±10.3
Range	15–83	17–88	19–68
**Body mass index**			
Mean ± SD	23.0±4.2	23.9±4.5	
Range	13–41	15–54	
**Age at diagnosis (yrs)**			
Mean ± SD	26.1±12.3	28.9±14.5	
Range	1–78	2–81	
**Disease duration (yrs)**			
Mean ± SD	13.4±8.9	12.2±8.3	
Range	0–47	1–50	
**Positive family history of IBD (%)**	16.8	17.4	

### DNA extraction and genotyping of the IL12B variants

From all study participants, blood samples were taken and genomic DNA was isolated from peripheral blood leukocytes using the DNA blood mini kit from Qiagen (Hilden, Germany) according to the manufacturer's guidelines. Four *IL12B* SNPs (rs3212227, rs17860508, rs10045431 and rs6887695) were genotyped by PCR and melting curve analysis using a pair of fluorescence resonance energy transfer (FRET) probes in a LightCycler® 480 Instrument (Roche Diagnostics, Mannheim, Germany) as described in previous studies [6], [40], [42], [43]. These *IL12B* SNPs were selected based on previous studies showing associations with CD or other autoimmune diseases such as psoriasis. Specifically, the SNPs rs3212227 and rs6887695 were selected from the study of Cargill and co-workers [29], while rs6887695 was also investigated in the study of Parkes *et al.* [8]. The SNP rs10045431 was selected from the study of Barrett and co-workers [5]. Additionally, the SNP rs17860508 located within the promoter of *IL12B* was included because of its potential functional relevance regarding gene expression [44]. The total volume of the PCR was 5 µl containing 25 ng of genomic DNA, 1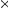 Light Cycler 480 Genotyping Master Mix (Roche Diagnostics), 2.5 pmol of each primer and 0.75 pmol of each FRET probe (TIB MOLBIOL, Berlin, Germany). In the case of rs3212227 and rs10045431, the concentrations of the forward primers and in the case of rs17860508 the concentration of the reverse primer was reduced to 0.5 pmol. In the case of rs6887695 the concentration of the reverse primer was reduced to 1.25 pmol and in the case of rs744166 the concentration of the forward primer was reduced to 0.75 pmol, respectively. Two SNPs were analyzed in a multiplex reaction, the combinations were rs3212227+rs17860508 and rs10045431+rs6887695. The PCR comprised an initial denaturation step (95°C for 10 min) and 45 cycles (95°C for 10°C sec, 60 for 10 sec, 72°C for 15 sec). The melting curve analysis comprised an initial denaturation step (95°C for 1 min), a step rapidly lowering the temperature to 40°C and holding for 2 min, and a heating step slowly (1 acquisition/°C) increasing the temperature up to 95°C and continuously measuring the fluorescence intensity. The results of the melting curve analysis were confirmed by analyzing two patient samples for each possible genotype using sequence analysis. For sequencing, the total volume of the PCR was 100 µl containing 250 ng of genomic DNA, 1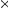 PCR-buffer (Qiagen, Hilden, Germany), a final MgCl_2_ concentration of 2 mM, 0.5 mM of a dNTP mix (Sigma, Steinheim, Germany), 2.5 units of HotStar Plus Taq™ DNA polymerase (Qiagen) and 10 pmol of each primer (TIB MOLBIOL). The PCR comprised an initial denaturation step (95°C for 5 min), 35 cycles (denaturation at 94°C for 30 sec, primer annealing at 65°C for 30 sec, extension at 72°C for 30 sec) and a final extension step (72°C for 10 min). The PCR products were purified using the QIAquick PCR Purification Kit (Qiagen) and sequenced by a commercial sequencing company (Sequiserve, Vaterstetten, Germany). All sequences of primers and FRET probes and primer annealing temperatures used for genotyping and for sequence analysis, respectively, are given in Supplementary Tables S1 and S2.

### STAT4 and IL23R genotyping

For analysis of epistatic interactions, seven *STAT4* SNPs (rs11889341, rs7574865, rs7568275, rs8179673, rs10181656, rs7582694, rs10174238) and 10 *IL23R* SNPs (rs1004819, rs7517847, rs10489629, rs2201841, rs11465804, rs11209026 = p.Arg381Gln, rs1343151, rs10889677, rs11209032, rs1495965) were genotyped by PCR and melting curve analysis using a pair of fluorescence resonance energy transfer (FRET) probes in a LightCycler® 480 Instrument (Roche Diagnostics, Mannheim, Germany) as described previously [6], [40]. The majority of *IL23R* and *STAT4* genotype data were available from previous studies [6], [40].

### In silico analysis of transcription factor binding sites

SNPs rs3212227, rs17860508, rs10045431 and rs6887695 were analyzed for potential transcription factor binding sites applying the online tool TFSEARCH (http://www.cbrc.jp/research/db/TFSEARCH.html). It is based on the TRANSFAC database developed at GBF Braunschweig, Germany [45]. The threshold score for binding sites was set to 75.0 (score = 100.0 * (‘weighted sum’−min)/(max−min); max. score = 100). For each SNP, major and minor alleles including the flanking sequences 15 bp upstream and downstream were analyzed. Only human transcription factors were included in the analysis.

### Statistical analyses

Power calculation was performed using the Genetic Power Calculator (http://pngu.mgh.harvard.edu/~purcell/gpc/). Each genetic marker was tested for Hardy-Weinberg equilibrium in the control population. For data evaluation, we used PLINK (http://pngu.mgh.harvard.edu/~purcell/plink/) and R-2.13.1. (http://cran.r-project.org). Haplotypes tests were performed in a sliding-window approach including up to 4 markers. Epistasis between different SNPs was tested using the –epistasis option on PLINK. Genotype-phenotype associations were assessed using logistic regression.

## Results

### The IL12B polymorphisms rs6887695 is associated with increased IBD susceptibility

In all three subgroups (CD, UC, and controls), the allele frequencies of the *IL12B* SNPs (rs3212227, rs17860508, rs10045431, rs6887695) were in accordance with the predicted Hardy-Weinberg equilibrium (Table 2). The *IL12B* SNP rs6887695 showed an association with increased IBD susceptibility (p = 0.035; OR 1.15 [95% CI 1.01–1.31]). In addition, there was a trend for rs6887695 for association with CD (OR 1.41 [95% CI 0.99–1.31], p = 0.066) and UC (OR 1.18 [95% CI 0.97–1.43], p = 0.092) and a trend for association of rs10045431 with UC (OR 0.83 [95% 0.68–1.02], p = 0.083; Table 2).

**Table 2:** Associations of *IL12B* gene markers in CD and UC case-control association studies.

SNP	Minor allele	Crohn's disease*n = 913*			Ulcerative colitis*n = 318*			Inflammatory bowel disease*n = 1231*			Controls*n = 965*
		MAF	p value	OR [95% CI]	MAF	p value	OR [95% CI]	MAF	p value	OR [95% CI]	MAF
rs3212227	G	0.209	0.684	0.97 [0.82–1.13]	0.214	0.777	1.03 [0.83–1.29]	0.206	0.820	0.98 [0.85–1.14]	0.209
rs17860508	TTAGAG	0.485	0.974	1.00 [0.88–1.14]	0.489	0.854	1.02 [0.85–1.22]	0.486	0.903	1.01 [0.89–1.14]	0.484
rs10045431	A	0.271	0.258	0.92 [0.80–1.06]	0.252	*0.083*	0.83 [0.68–1.02]	0.266	0.109	0.90 [0.78–1.02]	0.288
rs6887695	C	0.329	*0.066*	1.41 [0.99–1.31]	0.336	*0.092*	1.18 [0.97–1.43]	0.330	**0.035**	1.15 [1.01–1.31]	0.300

The category “Inflammatory bowel disease” represents the combined CD and UC cohort. Minor allele frequencies (MAF), allelic test *P*-values, and odds ratios (OR, shown for the minor allele) with 95% confidence intervals (CI) are depicted for both the CD and UC case-control cohorts. Significant associations (p<0.05) are highlighted in **bold fonts**, suggestive associations (p<0.10) are depicted in *Italic fonts*.

### IL12B haplotype analysis

We next performed a detailed haplotype analysis in our IBD cohort. As shown in Tables 3 and 4, we could not demonstrate significant associations of *IL12B* haplotypes with CD and UC susceptibility. A haplotype of all four investigated SNPs (rs3212227-rs17860508-rs10045431-rs6887695) showed a trend for association with CD (p = 0.053); however, this potential association signal did not reach statistical significance and was uncorrected for multiple testing.

**Table 3:** Haplotypes of *IL12B* SNPs in the CD case-control sample and omnibus p-values for association with CD susceptibility.

Haplotype combination	Omnibus p-value
rs3212227-rs17860508	0.957
rs17860508-rs10045431	0.333
rs10045431-rs6887695	0.168
rs3212227-rs17860508-rs10045431	0.219
rs17860508-rs10045431-rs6887695	0.202
rs3212227-rs17860508-rs10045431-rs6887695	*0.053*

*P*-values showing a trend towards significance are depicted in *Italic fonts*.

**Table 4:** Haplotypes of *IL12B* SNPs in the UC case-control sample and omnibus p-values for association with UC susceptibility.

Haplotype combination	Omnibus p-value
rs3212227-rs17860508	0.614
rs17860508-rs10045431	0.319
rs10045431-rs6887695	0.126
rs3212227-rs17860508-rs10045431	0.288
rs17860508-rs10045431-rs6887695	0.206
rs3212227-rs17860508-rs10045431-rs6887695	0.300

### Genotype-phenotype analysis: C/C homozygosity for the SNP rs6887695 is associated with non-stricturing, non-penetrating Crohn's disease

The majority of previous GWAS meta-analyses showing an association with *IL12B* were performed in CD patients with predominant ileal involvement [5], [9]. To exclude that only a certain IBD subphenotype such as ileal CD is associated with *IL12B*, we further investigated potential associations of *IL12B* SNPs with the anatomic location in IBD patients Genotype-phenotype analysis showed a weak association of rs3212227 with colonic CD (p = 0.04) but not with ileal involvement. In addition, we analyzed the *IL12B* SNP rs6887695, for which we found a trend for association with CD and UC, regarding potential phenotypic consequences in CD and UC. CD patients, who were homozygous C/C carriers of this SNP, had significantly more often a non-stricturing, non-penetrating disease phenotype than carriers of the G allele ( = combined group of heterozygous C/G and wildtype G/G carriers; p = 6.8×10^−5^ (Table 5). This strong association remained significant following Bonferroni correction for multiple testing. C/C homozygous carriers of the *IL12B* SNP rs6887695 had also less stenoses than G carriers (p = 0.038) and there was a trend towards less penetrating disease (p = 0.062), less fistulas (p = 0.085), and less surgery (p = 0.063; Table 5). In UC, homozygous C/C carriers of the *IL12B* SNP rs6887695 had significantly more often left-sided UC (p = 0.006) but less often extensive UC (p = 0.029) than the combined group of heterozygous C/G and wildtype G/G carriers (Table 6).

**Table 5:** Genotype-phenotype-analysis of SNP rs6887695 in patients with Crohn's disease (CD).

*IL12B*	(1) CC	(2) CG	(3) GG	P_CC_	OR_CC_
**rs6887695**	*n = 100*	*n = 394*	*n = 409*		**[95% CI]**
**Location**					
*(n = 768)*	*n = 80*	*n = 331*	*n = 357*		
Terminal ileum	11	55	46	1.000	0.92
(L1)	(13.8%)	(16.6%)	(12.9%)		[0.45–1.88]
Colon	6	43	49	0.158	0.53
(L2)	(7.5%)	(13.0%)	(13.7%)		[0.20–1.30]
Ileocolon	60	230	258	0.514	1.23
(L3)	(75.0%)	(69.5%)	(72.3%)		[0.70–2.17]
Upper GI	3	3	4	*0.076*	3.79
(L4)	(3.8%)	(0.9%)	(1.1%)		[0.76–16.69]
**Behaviour** 1					
*(n = 690)*	*n = 72*	*n = 293*	*n = 325*		
Non-stricturing & Non-penetrating	31	65	75	6.8×10^−5^*	2.84
(B1)	(43.1%)	(22.2%)	(23.1%)		[1.66–4.84]
Stricturing(B2)	14(19.4%)	87(29.7%)	85(26.2%)	0.160	0.63[0.32–1.19]
Penetrating	27	141	165	*0.062*	0.61
(B3)	(37.5%)	(48.1%)	(50.8%)		[0.36–1.04]
					0.36
				9.5×10^−5^*	[0.21–0.62]
				**(B2+B3)**	(B2+B3)
**Use of immuno-suppressive agents** 2	50	202	225	0.604	0.84
*(n = 477/583)*	(79.7%)	(82.1%)	(82.1%)		[0.42–1.69]
**Surgery because of CD** 3	35	188	200	*0.063*	0.63
*(n = 423/808)*	(42.2%)	(52.9%)	(54.2%)		[0.39–1.027]
**Fistulas**	33	172	193	*0.085*	0.67
*(n = 398/825)*	(39.3%)	(47.8%)	(50.7%)		[0.41–1.08]
**Stenosis**	42	221	225	**0.038**	0.62
*(n = 488/827)*	(48.3%)	(61.1%)	(59.5%)		[0.39–0.98]

P_CC:_ *P*-value for testing for differences between homozygous carriers of the C allele (C/C) and heterozygous and non-carriers of the C allele. OR_CC_: corresponding odds ratios and 95% confidence intervals (95% CI). Significant *P*-values (<0.05) are depicted in **bold**, *P*-values showing a trend towards significance are depicted in *Italic fonts*. P-values marked with an asterisk * remained significant after Bonferroni correction.

1 Disease behaviour was defined according to the Montreal classification. A stricturing disease phenotype was defined as presence of stenosis without penetrating disease. The diagnosis of stenosis was made surgically, endoscopically, or radiologically (using MRI enteroclysis).

2 Immunosuppressive agents included azathioprine, 6-mercaptopurine, 6-thioguanin, methotrexate, infliximab and/or adalimumab.

3 Only surgery related to CD-specific problems (e.g. fistulectomy, colectomy, ileostomy) was included.

**Table 6:** Genotype-phenotype-analysis of SNP rs6887695 in patients with ulcerative colitis (UC) for which detailed phenotypic data based on the Montreal classification was available.

*IL12B*	(1) CC	(2) CG	(3) GG	P_CC_	OR_CC_
**rs6887695**	*n = 34*	*n = 145*	*n = 138*		**[95% CI]**
**Location**					
*(n = 193)*	*n = 25*	*n = 94*	*n = 74*		
**Proctitis (E1)**	3	10	11	1.000	0.96
	(12.0%)	(10.6%)	(14.9%)		[0.21–3.78]
				**0.030**	[1.07–7.03]
				**(E1+E2)**	(E1+E2)
**Left-sided UC (E2)**	9	10	11	0.006*	3.94
	(36.0%)	(10.6%)	(14.9%)		[1.40–11.02]
**Extensive UC (E3)**	13	74	52	**0.029**	0.36
	(52.0%)	(78.7%)	(70.3%)		[0.14–0.92]
**Extra-intestinal manifestations**	3	26	24	0.747	0.66
*(n = 53/149)*	(27.3%)	(35.1%)	(37.5%)		[0.13–2.91]
**Use of immunosuppressive agents**	22	97	82	0.818	1.23
*(n = 201/267)*	(78.6%)	(77.6%)	(71.9%)		[0.45–3.57]
**Abscesses**	2	6	4	0.365	1.77
(*n = 12*/240)	(7.7%)	(5.4%)	(3.9%)		[0.25–9.41]

For each variable, the number of patients included is given. P_CC_ *P*-value for testing for differences between homozygous carriers of the C allele and heterozygous/non-carriers of the C allele. OR_CC_: corresponding odds ratios and 95% confidence intervals (95% CI). Significant *P*-values (<0.05) are depicted in **bold fonts**. *P*-values marked with an asterisk remained significant following Bonferroni correction.

### Analysis for epistasis of IL12B with IL23R and STAT4 gene variants regarding susceptibility to Crohn's disease

Next, we analyzed potential evidence for epistasis of *IL12B* variants with other CD susceptibility genes involved in IL-12 and Th17 signaling, including CD-associated variants in the *IL23R* and *STAT4* gene [3], [6], [40]. Analysis for gene-gene interaction revealed weak epistasis of *IL12B* SNP rs6887695 with 7 out of the 8 analyzed *STAT4* gene variants (p<0.05) regarding CD susceptibility (Table 7). However, following Bonferroni correction, none of these associations remained significant. In addition, there was no evidence for epistasis between *IL23R* and *IL12B* variants (Table 7).

**Table 7:** Analysis for gene-gene interaction of *IL12B* with *IL23R* and *STAT4* variants, respectively, regarding susceptibility to Crohn's disease (CD).

*IL12B* SNPs	rs3212227	rs17860508	rs10045431	rs6887695
***IL23R*** ** SNPs**				
rs1004819	0.408	0.731	0.474	0.450
rs7517847	0.902	0.895	0.743	0.956
rs10489629	0.577	0.284	0.757	0.561
rs2201841	0.497	0.428	0.080	0.189
rs11465804	0.793	0.852	0.819	0.110
rs11209026 = p.Arg381Gln	0.280	0.752	0.929	0.181
rs1343151	0.970	0.745	0.874	0.971
rs10889677	0.485	0.688	0.108	0.120
rs11209032	0.311	0.473	0.890	0.427
rs1495965	0.641	0.640	0.518	0.624
***STAT4*** ** SNPs**				
rs11889341	0.732	0.092	0.160	**0.025**
rs7574865	0.845	0.069	0.223	**0.031**
rs7568275	0.725	0.115	0.229	**0.045**
rs8179673	0.659	0.120	0.160	**0.035**
rs10181656	0.618	0.115	0.230	**0.040**
rs7582694	0.818	0.085	0.162	**0.021**
rs10174238	0.354	0.200	0.625	0.247

Significant associations are highlighted in **bold fonts**. None of the associations remained significant following Bonferroni correction.

### In silico analysis of IL12B SNPs identifies potential differences in transcription factor binding to major and minor alleles

To analyze if SNPs in the *IL12B* region modify the binding of transcription factors to DNA and thereby modulating gene expression, we analyzed SNPs rs3212227, rs17860508, rs10045431 and rs6887695 including the surrounding sequences for potential binding sites as described in the methods section. The results are shown in table 8. Interestingly, the highest differences in binding scores for the major and the minor alleles was found for rs6887695, the only SNP that was associated with overall IBD susceptibility in our study cohort. Moreover, in homozygous carriers of the minor allele of rs6887695, there were significant associations with specific CD and UC phenotypes. While the transcription factors HSF1, HSF2, MZF1 and Oct-1 were predicted to bind with very high probability to the sequence comprising the major G allele, predicted binding to the minor C allele was substantially lower (Table 8). On the other hand, the binding score for the transcription factor RORα was higher for the minor C allele (Table 8). Therefore, differential DNA binding of transcription factors in this genomic region followed by differential gene transcription might be one reason for the disease-modifying abilities of this SNP that we observed in our genotype-phenotype analysis.

**Table 8:** Overview of potential transcription factor binding sites in the genomic regions harboring the analyzed *IL12B* SNPs rs6887695, rs10045431, rs17860508, and rs3212227.

*IL12B* SNP	Factor	Consensus sequence‡	position relative to SNP (5′ to 3′)	Binding score major allele	Binding score minor allele
**rs6887695** (approx. 65 kb upstream of *IL12B*):					
GTGTAGTGTAGTGGT **[** ***C/G*** **]** AATAGTCTGGATTTA					
	HSF2	NGAANNWTCK	−1 to +8	***85.9***	***68.6***
	AML-1a	TGCGGT	−6 to −1	85.4	85.4
	MZF1	NGNGGGGA	−6 to +1	***84.3***	***67.0***
	Oct-1	NNGAATATKCANNNN	−5 to +9	***82.5***	***70.6***
	AML-1a	TGCGGT	−14 to −9	81.4	81.4
	AML-1a	TGCGGT	−9 to −4	81.4	81.4
	Th1/E4	NNNNGNRTCTGGMWTT	−1 to +14	80.4	80.8
	HSF1	RGAANRTTCN	−1 to +8	***79.0***	***62.7***
	RORα	NWAWNNAGGTCAN	−10 to +2	***64.4***	***75.9***
**rs10045431** (approx. 57 kb upstream of *IL12B*):					
GCAGGCACAGCCCAG **[** ***A/C*** **]** ATTAAACTCTCAAAT					
	Oct-1	CWNAWTKWSATRYN	−2 to +11	79.6	75.5
	SRY	AAACWAM	+4 to +10	77.3	77.3
	Pbx-1	ANCAATCAW	−2 to +6	**75.5**	**69.6**
	Sp1	GRGGCRGGGW	−11 to −2	75.3	75.3
	Tst-1/Oct-6	NNKGAWTWANANTKN	−4 to +10	***70.8***	***81.2***
**rs17860508** * (approx. 2.7 kb upstream of *IL12B*):					
AATGTGGGGGCCACA **[** ***-/G/GC/TTAGA/TTAGAG*** **]** CCTCTCTCGGAGACA					
	GATA-3^&^	NNGATARGN	0 to +9	**82.5**	**74.1**
	AML-1a	TGCGGT	−6 to −1	83.7	83.7
	AML-1a	TGCGGT	−13 to −8	82.7	82.7
	MZF1	NGNGGGGA	−13 to −6	80.9	80.9
	Oct1^§^	NNRTAATNANNN	−6 to +1	79.3	77.5
	Sp1^#^	GRGGCRGGGW	−7 to +3	***75.3***	***61.6***
**rs3212227** (3′-UTR of *IL12B*):					
TGTATTTGTATAGTT **[** ***A/C*** **]** GATGCTAAATGCTCA					
	C/EBP	NNTKTGGWNANNN	−13 to −1	87.7	87.7
	Brn-2/Oct-3	NNCATNSRWAATNMRN	−1 to +16	85.1	83.2
	TATA	NCTATAAAAR	−11 to −2	84.7	84.7
	SRY	AAACWAM	−10 to −4	80.9	80.9
	Oct-1	TNTATGNTAATT	−1 to +10	80.2	79.6
	Tst-1/Oct-6	NNKGAWTWANANTKN	0 to +14	79.2	77.1
	CDP CR	NATYGATSSS	−3 to +6	**78.5**	**73.1**
	Oct-1	CWNAWTKWSATRYN	−7 to +6	**77.6**	**83.7**
	SRY	AAACWAM	−15 to −9	77.3	77.3
	C/EBP	NNTKTGGWNANNN	−1 to +10	76.2	76.9
	CDP CR	NATYGATSSS	−5 to +4	75.3	73.1
	SRY	NWWAACAAWANN	−9 to +2	71.8	76.2

Binding scores differing more than 5 points between major and minor alleles are depicted in **bold**. Scores differing more than 10 points are depicted in ***bold italic***. The binding score threshold for each allele was set to 75.0.

‡ Different consensus sequences for the same transcription factor are caused by the deduction of the sequences from different matrices in the TRANSFAC database [45].

* For rs17860508, more than two alleles exist. For the major allele, the score comprises the highest score from all four non-minor alleles (-, G, GC, TTAGA). & = score for the GC allele; # = score for the (-) allele; § = score for the TTAGA allele.

UTR = untranslated region;

Nucleotide codes: K = G or T, M = A or C, R = A or G, S = C or G, W = A or T, N = A, G, C or T.

## Discussion

This study presents a detailed genotype-phenotype analysis investigating *IL12B* SNPs as potential susceptibility gene variants in a large Caucasian IBD cohort. A major focus of this study was the analysis of potential epistatic interactions with key gene variants of the IL-12 and IL-23/Th17 pathway such as *IL23R* and *STAT4*. We demonstrated that the *IL12B* variant rs6887695 is weakly associated with overall IBD susceptibility (p = 0.035), with trends for association with both CD and UC susceptibility. Moreover, a haplotype block formed by the four investigated *IL12B* SNPs showed a trend for association with CD (p = 0.05). Considering our findings and the strong association signal for this gene in the recent GWAS [9], [10], *IL12B* can be regarded as established IBD susceptibility gene. In contrast to the very large IBD GWAS, our sample size (n = 2196) was smaller, resulting in limited power, particularly for the UC cohort. For example, a power calculation, which assumed an OR of 1.2 and an allele frequency of 0.20 for the rarest *IL12B* variant rs3212227, demonstrated that our study had 63.1% power to detect a nominal significant finding (alpha = 0.05) in the CD cohort but had only a power of 38.2% in the UC cohort. However, based on the results of this study, the strength of a potential association of *IL12B* with IBD is several log-fold weaker than that shown for *IL23R* in our cohort [6] and other major CD susceptibility genes such as *NOD2* and *ATG16L1* (Supplemental Table S3), suggesting a more important role for IL-23R than IL-12/23 p40 in the genetic susceptibility to IBD. This is in agreement with the recent IBD meta-analyses [9], [10], which showed much stronger IBD association signals for *IL23R* than for *IL12B*. Similarly, smaller studies failed to show an association of *IL12B* SNPs with IBD or showed only weak associations with CD or UC [46], [47], [48] (see Supplemental Table S4 for an overview on published studies on *IL12B* in IBD). Even large studies such as the study by Festen et al., which included 1,455 UC patients and 1,902 controls, was unable to show an association of *IL12B* with UC [49], further demonstrating that very large patient cohorts are necessary to show convincing associations with this gene locus. Therefore, *IL23R* is a more important genetic modifier of IBD susceptibility than *IL12B*, suggesting a more important pathogenic role of Th17 cells, which express IL-23R, than Th1 cells, which develop under the control of IL-12.

Currently, there are limited data on how *IL12B* may functionally influence IBD susceptibility in humans. A very recent study in mice demonstrated that a polymorphism in the coding region of murine *Il12b* promotes IL-12p70 and IL-23 heterodimer formation [50]. The authors hypothesized that the high synthesis rate of IL-12/23 cytokines resulting from efficient binding may lead to rapid proinflammatory skewing of immune responses and disturbance of the homeostatic balance resulting in higher susceptibility for IBD [50]. Other gene variants may also modulate IL-12 expression as recently demonstrated by our group showing increased basal levels of IL-12p40 in CD patients with two mutated *NOD2* alleles [51]. Currently, it is unclear which *IL12B* SNP is the “true” disease-causing variant. We therefore performed a detailed *in silico* analysis of *IL12B* regarding potential transcription factor binding sites and demonstrated for rs6887695 (out of the four analyzed *IL12B* SNPs) the highest likelihood of changes in transcription factor binding. This is consistent with the results of our genotype analysis in which only rs6887695 was associated with overall IBD susceptibility and potential epistasis with *STAT4* SNPs.

For rs6887695, the *in silico* analysis predicted changes in the binding of the transcription factors HSF1, HSF2, MZF1, Oct-1 and RORα. HSF1 plays a role in protecting against DSS-induced colitis [52]. MZF1 and Oct-1 are transcriptional regulators of SERPINA3 [53], [54] which has been implicated as one of the epithelium-derived genes involved in the antibacterial defense [55]. In addition, our *in silico* analysis predicted for the transcription factor RORα stronger binding to the minor C allele. Interestingly, RORα plays together with RORγt a key role in the development of Th17 cells which are involved in the pathogenesis of CD and UC [1] and may explain the association of rs6887695 with increased IBD susceptibility found in our study. Although Th17 cells are recognized as proinflammatory T cell population, their major effector cytokines IL-17A and IL-22 may have also protective functions under certain circumstances [19], [56], [57], [58], [59]. Similarly, studies indicated for RORα not only proinflammatory effects but also a role as negative regulator of inflammatory responses [60]. For example, RORα inhibits TNF-α-induced IL-6, IL-8 and COX-2 expression in human primary smooth-muscle cells by negatively interfering with the NF-κB signaling pathway and reducing p65 translocation [60]. This may correlate with our genotype-phenotype analysis which demonstrated a less severe disease phenotype for homozygous C/C carriers of the *IL12B* SNP rs6887695. CD patients, who were homozygous C/C carriers of this SNP, had significantly more often non-stricturing, non-penetrating disease than carriers of the G allele (p = 6.8×10^−5^), while C/C homozygous UC patients had less often extensive colitis than G allele carriers (p = 0.029).

*IL12B* is another example for a common susceptibility gene for CD and UC which is supported by the results of our study showing a trend for rs6887695 for association with both CD and UC. Out of 99 currently known IBD susceptibility gene loci (71 in CD and 47 in UC), at least 28 susceptibility loci are shared between CD and UC [9], [10]. Remarkably, the strongest cluster of common CD and UC susceptibility genes is formed by genes related to the IL-23/Th17 pathway including *IL12B* [61]. Similar to *IL23R*, *IL12B* is also a shared susceptibility gene with other IBD-associated diseases such as psoriasis [29] and ankylosing spondylitis [28], providing an explanation of the increased incidence of these extraintestinal manifestations in IBD patients.

The initial GWAS focused on CD patients with ileal CD. To exclude that the associations found in recent GWAS meta-analysis are based only on certain IBD subphenotypes, we performed a detailed genotype-phenotype analysis focusing also on the anatomic disease localization. Interestingly, genotype-phenotype analysis showed an association of *IL12B* rs3212227 with colonic CD but not with ileal CD. It is likely that many of the susceptibility loci, which are shared by CD and UC, may predispose to a common phenotype such as colonic (and not ileal) IBD. This is supported by the association of the *IL12B* SNP rs3212227 with colonic CD and the trend for an association signal of rs6887695 with both CD and UC susceptibility found in our study. Similarly, in another study from New Zealand, carriers of the minor C allele of the *IL12B* SNP rs6887695 had a decreased risk of ileal disease [48]. A recent study analyzing the same SNP (rs6887695) suggested additional environmental triggers regarding the risk of this *IL12B* SNP on CD susceptibility. Analyzing differences in associated genes between smoking and non-smoking CD patients, it implicated a complex gene-environment interaction demonstrating an association of *IL12B* SNP rs6887695 in non-smoking, but not in smoking patients [62].

In addition, our analysis of potential gene-gene interactions revealed weak epistasis of *IL12B* SNP rs6887695 with 7 out of 8 analyzed *STAT4* gene variants (p<0.05). This is highly interesting, given that STAT4 is the major downstream transcription factor of IL-12. However, considering our limited sample size and the borderline significance of this interaction, which was lost after Bonferroni correction, this potential gene-gene interaction needs further analysis in very large cohorts or GWAS meta-analyses. However, in contrast to our hypothesis-driven study, which analyzed SNPs in the *IL12B* gene encoding IL-12 p40 and the gene *STAT4* encoding the major IL-12 downstream transcription factor STAT4, GWAS are limited by the problem of multiple testing to a much greater extent than our study. Given the large number of SNPs analyzed in GWAS, the correction factor (to correct for multiple testing) is much higher than in our study, which may result in the elimination of potentially true gene-gene interactions. Moreover, in the study by Anderson et al. no formal testing for epistasis has been performed [10]. In addition, in the manuscript by Franke et al. [9], only epistasis between the 71 significantly associated CD loci, which did not include *STAT4*, was analyzed.

In summary, in contrast to Th17 cell-modifying and strongly IBD-associated *IL23R* gene variants, *IL12B* variants have a lesser role in the susceptibility to CD and UC in the German population, suggesting a more important role of IL-23R-expressing Th17 cells than Th1 cells in the CD pathogenesis. This observation is supported by similar results of large GWAS [5], [9], [10], in which *IL23R* showed stronger associations with IBD susceptibility than *IL12B*, although these GWAS clearly established *IL12B* as IBD susceptibility gene. Our *IL12B in silico* analysis and the results of our genotype analysis suggest rs6887695 as likely disease-causing *IL12B* variant. Homozygous C/C carriers of this SNP were protected against stricturing and penetrating CD and showed less often extensive UC which may be related to the alteration of the binding of certain transcription factors such as RORα as predicted by our *in silico* analysis. We demonstrated for rs688769 potential epistasis with *STAT4*, encoding the major downstream transcription factor of IL-12 which would link IL-12 and STAT4 for the first time on a genetic level. Moreover, similar to an association of the *STAT4* SNP rs7574865 with colonic CD [40], we found an association of the *IL12B* SNP rs3212227 with an increased risk for colonic CD. Based on the gene-gene interaction found for *IL12B* and *STAT4* in this study, large meta-analyses (e.g., by the International IBD Genetics Consortium), which may include our data should further investigate potential epistasis between the main IBD susceptibility genes as major pathomechanism in the disease pathogenesis. Further fine mapping and functional studies are required to clarify the “true” disease-causing *IL12B* variant and its pathogenic role in IBD susceptibility.

## Supporting Information

Table S1 Primer sequences (F: forward primer, R: reverse Primer), FRET probe sequences, and primer annealing temperatures used for genotyping of *IL12B* variants. Note: FL: Fluorescein, LC610: LightCycler-Red 610; LC640: LightCycler-Red 640; LC670: LightCycler-Red 670. The polymorphic position within the sensor probe is underlined. A phosphate is linked to the 3′-end of the acceptor probe to prevent elongation by the DNA polymerase in the PCR given based on a median split.
(DOC)

Table S2 Primer sequences used for the sequence analysis of the *IL12B* variants.
(DOC)

Table S3 Comparison of the association signals of *IL12B* with the association signals of the three most strongly CD-associated genes *NOD2*, *IL23R* and *ATG16L1* in the Munich IBD case-control cohort. Minor allele frequencies (MAF), allelic test *P*-values, and odds ratios (OR, shown for the minor allele) with 95% confidence intervals (CI) are depicted for both the CD and UC case-control cohorts. Details on the analyses of *NOD2*, *IL23R* and *ATG16L1* gene variants were published in previous studies.
(DOC)

Table S4 Overview of published studies on *IL12B* in patients with inflammatory bowel diseases. CD: Crohn's disease; UC: ulcerative colitis.
(DOC)

## References

[1] Brand S (2009). Crohn's disease: Th1, Th17 or both? The change of a paradigm: new immunological and genetic insights implicate Th17 cells in the pathogenesis of Crohn's disease.. Gut.

[2] Neurath MF (2007). IL-23: a master regulator in Crohn disease.. Nat Med.

[3] Duerr RH, Taylor KD, Brant SR, Rioux JD, Silverberg MS (2006). A genome-wide association study identifies IL23R as an inflammatory bowel disease gene.. Science.

[4] Abraham C, Cho J (2009). Interleukin-23/Th17 pathways and inflammatory bowel disease.. Inflamm Bowel Dis.

[5] Barrett JC, Hansoul S, Nicolae DL, Cho JH, Duerr RH (2008). Genome-wide association defines more than 30 distinct susceptibility loci for Crohn's disease.. Nat Genet.

[6] Glas J, Seiderer J, Wetzke M, Konrad A, Torok HP (2007). rs1004819 is the main disease-associated IL23R variant in German Crohn's disease patients: combined analysis of IL23R, CARD15, and OCTN1/2 variants.. PLoS ONE.

[7] Rioux JD, Xavier RJ, Taylor KD, Silverberg MS, Goyette P (2007). Genome-wide association study identifies new susceptibility loci for Crohn disease and implicates autophagy in disease pathogenesis.. Nat Genet.

[8] Parkes M, Barrett JC, Prescott NJ, Tremelling M, Anderson CA (2007). Sequence variants in the autophagy gene IRGM and multiple other replicating loci contribute to Crohn's disease susceptibility.. Nat Genet.

[9] Franke A, McGovern DP, Barrett JC, Wang K, Radford-Smith GL (2010). Genome-wide meta-analysis increases to 71 the number of confirmed Crohn's disease susceptibility loci.. Nat Genet.

[10] Anderson CA, Boucher G, Lees CW, Franke A, D'Amato M (2011). Meta-analysis identifies 29 additional ulcerative colitis risk loci, increasing the number of confirmed associations to 47.. Nat Genet.

[11] Glas J, Stallhofer J, Ripke S, Wetzke M, Pfennig S (2009). Novel genetic risk markers for ulcerative colitis in the IL2/IL21 region are in epistasis with IL23R and suggest a common genetic background for ulcerative colitis and celiac disease.. Am J Gastroenterol.

[12] Wang K, Zhang H, Kugathasan S, Annese V, Bradfield JP (2009). Diverse genome-wide association studies associate the IL12/IL23 pathway with Crohn Disease.. Am J Hum Genet.

[13] Langrish CL, McKenzie BS, Wilson NJ, de Waal Malefyt R, Kastelein RA (2004). IL-12 and IL-23: master regulators of innate and adaptive immunity.. Immunol Rev.

[14] Chan SH, Perussia B, Gupta JW, Kobayashi M, Pospisil M (1991). Induction of interferon gamma production by natural killer cell stimulatory factor: characterization of the responder cells and synergy with other inducers.. J Exp Med.

[15] Parronchi P, Romagnani P, Annunziato F, Sampognaro S, Becchio A (1997). Type 1 T-helper cell predominance and interleukin-12 expression in the gut of patients with Crohn's disease.. Am J Pathol.

[16] Oppmann B, Lesley R, Blom B, Timans JC, Xu Y (2000). Novel p19 protein engages IL-12p40 to form a cytokine, IL-23, with biological activities similar as well as distinct from IL-12.. Immunity.

[17] Ciric B, El-behi M, Cabrera R, Zhang GX, Rostami A (2009). IL-23 drives pathogenic IL-17-producing CD8+ T cells.. J Immunol.

[18] Seiderer J, Elben I, Diegelmann J, Glas J, Stallhofer J (2008). Role of the novel Th17 cytokine IL-17F in inflammatory bowel disease (IBD): upregulated colonic IL-17F expression in active Crohn's disease and analysis of the IL17F p.His161Arg polymorphism in IBD.. Inflamm Bowel Dis.

[19] Brand S, Beigel F, Olszak T, Zitzmann K, Eichhorst ST (2006). IL-22 is increased in active Crohn's disease and promotes proinflammatory gene expression and intestinal epithelial cell migration.. Am J Physiol Gastrointest Liver Physiol.

[20] Dambacher J, Beigel F, Zitzmann K, De Toni EN, Goke B (2009). The role of the novel Th17 cytokine IL-26 in intestinal inflammation.. Gut.

[21] Brand S, Olszak T, Beigel F, Diebold J, Otte JM (2006). Cell differentiation dependent expressed CCR6 mediates ERK-1/2, SAPK/JNK, and Akt signaling resulting in proliferation and migration of colorectal cancer cells.. J Cell Biochem.

[22] Fujino S, Andoh A, Bamba S, Ogawa A, Hata K (2003). Increased expression of interleukin 17 in inflammatory bowel disease.. Gut.

[23] Monteleone G, Monteleone I, Fina D, Vavassori P, Del Vecchio Blanco G (2005). Interleukin-21 enhances T-helper cell type I signaling and interferon-gamma production in Crohn's disease.. Gastroenterology.

[24] Parham C, Chirica M, Timans J, Vaisberg E, Travis M (2002). A receptor for the heterodimeric cytokine IL-23 is composed of IL-12Rbeta1 and a novel cytokine receptor subunit, IL-23R.. J Immunol.

[25] Sandborn WJ, Feagan BG, Fedorak RN, Scherl E, Fleisher MR (2008). A randomized trial of Ustekinumab, a human interleukin-12/23 monoclonal antibody, in patients with moderate-to-severe Crohn's disease.. Gastroenterology.

[26] Huffmeier U, Lascorz J, Bohm B, Lohmann J, Wendler J (2009). Genetic variants of the IL-23R pathway: association with psoriatic arthritis and psoriasis vulgaris, but no specific risk factor for arthritis.. J Invest Dermatol.

[27] Smith RL, Warren RB, Eyre S, Ho P, Ke X (2008). Polymorphisms in the IL-12beta and IL-23R genes are associated with psoriasis of early onset in a UK cohort.. J Invest Dermatol.

[28] Burton PR, Clayton DG, Cardon LR, Craddock N, Deloukas P (2007). Association scan of 14,500 nonsynonymous SNPs in four diseases identifies autoimmunity variants.. Nat Genet.

[29] Cargill M, Schrodi SJ, Chang M, Garcia VE, Brandon R (2007). A large-scale genetic association study confirms IL12B and leads to the identification of IL23R as psoriasis-risk genes.. Am J Hum Genet.

[30] Danoy P, Pryce K, Hadler J, Bradbury LA, Farrar C (2010). Association of variants at 1q32 and STAT3 with ankylosing spondylitis suggests genetic overlap with Crohn's disease.. PLoS Genet.

[31] Filer C, Ho P, Smith RL, Griffiths C, Young HS (2008). Investigation of association of the IL12B and IL23R genes with psoriatic arthritis.. Arthritis Rheum.

[32] Nair RP, Ruether A, Stuart PE, Jenisch S, Tejasvi T (2008). Polymorphisms of the IL12B and IL23R genes are associated with psoriasis.. J Invest Dermatol.

[33] Morahan G, Kaur G, Singh M, Rapthap CC, Kumar N (2007). Association of variants in the IL12B gene with leprosy and tuberculosis.. Tissue Antigens.

[34] Hirota T, Suzuki Y, Hasegawa K, Obara K, Matsuda A (2005). Functional haplotypes of IL-12B are associated with childhood atopic asthma.. J Allergy Clin Immunol.

[35] Tug E, Ozbey U, Tug T, Yuce H (2009). Relationship between the IL-12B promoter polymorphism and allergic rhinitis, familial asthma, serum total IgE, and eosinophil level in asthma patients.. J Investig Allergol Clin Immunol.

[36] Chang M, Saiki RK, Cantanese JJ, Lew D, van der Helm-van Mil AH (2008). The inflammatory disease-associated variants in IL12B and IL23R are not associated with rheumatoid arthritis.. Arthritis Rheum.

[37] Begovich AB, Chang M, Caillier SJ, Lew D, Catanese JJ (2007). The autoimmune disease-associated IL12B and IL23R polymorphisms in multiple sclerosis.. Hum Immunol.

[38] Marquez A, Mendoza JL, Taxonera C, Diaz-Rubio M, De La Concha EG (2008). IL23R and IL12B polymorphisms in Spanish IBD patients: no evidence of interaction.. Inflamm Bowel Dis.

[39] Yamazaki K, Takahashi A, Takazoe M, Kubo M, Onouchi Y (2009). Positive association of genetic variants in the upstream region of NKX2-3 with Crohn's disease in Japanese patients.. Gut.

[40] Glas J, Seiderer J, Nagy M, Fries C, Beigel F (2010). Evidence for STAT4 as a common autoimmune gene: rs7574865 is associated with colonic Crohn's disease and early disease onset.. PLoS ONE.

[41] Silverberg MS, Satsangi J, Ahmad T, Arnott ID, Bernstein CN (2005). Toward an integrated clinical, molecular and serological classification of inflammatory bowel disease: Report of a Working Party of the 2005 Montreal World Congress of Gastroenterology.. Can J Gastroenterol.

[42] Glas J, Konrad A, Schmechel S, Dambacher J, Seiderer J (2008). The ATG16L1 gene variants rs2241879 and rs2241880 (T300A) are strongly associated with susceptibility to Crohn's disease in the German population.. Am J Gastroenterol.

[43] Glas J, Seiderer J, Fries C, Tillack C, Pfennig S (2011). CEACAM6 gene variants in inflammatory bowel disease.. PLoS ONE.

[44] Muller-Berghaus J, Kern K, Paschen A, Nguyen XD, Kluter H (2004). Deficient IL-12p70 secretion by dendritic cells based on IL12B promoter genotype.. Genes Immun.

[45] Heinemeyer T, Wingender E, Reuter I, Hermjakob H, Kel AE (1998). Databases on transcriptional regulation: TRANSFAC, TRRD and COMPEL.. Nucleic Acids Res.

[46] Torkvist L, Halfvarson J, Ong RT, Lordal M, Sjoqvist U (2010). Analysis of 39 Crohn's disease risk loci in Swedish inflammatory bowel disease patients.. Inflamm Bowel Dis.

[47] Peter I, Mitchell AA, Ozelius L, Erazo M, Hu J (2011). Evaluation of 22 genetic variants with Crohn's disease risk in the Ashkenazi Jewish population: a case-control study.. BMC Med Genet.

[48] Ferguson LR, Han DY, Fraser AG, Huebner C, Lam WJ (2010). IL23R and IL12B SNPs and Haplotypes Strongly Associate with Crohn's Disease Risk in a New Zealand Population.. Gastroenterol Res Pract.

[49] Festen EA, Stokkers PC, van Diemen CC, van Bodegraven AA, Boezen HM (2010). Genetic analysis in a Dutch study sample identifies more ulcerative colitis susceptibility loci and shows their additive role in disease risk.. Am J Gastroenterol.

[50] Zwiers A, Fuss IJ, Seegers D, Konijn T, Garcia-Vallejo JJ (2011). A polymorphism in the coding region of Il12b promotes IL-12p70 and IL-23 heterodimer formation.. J Immunol.

[51] Beynon V, Cotofana S, Brand S, Lohse P, Mair A (2008). NOD2/CARD15 genotype influences MDP-induced cytokine release and basal IL-12p40 levels in primary isolated peripheral blood monocytes.. Inflamm Bowel Dis.

[52] Tanaka K, Namba T, Arai Y, Fujimoto M, Adachi H (2007). Genetic evidence for a protective role for heat shock factor 1 and heat shock protein 70 against colitis.. J Biol Chem.

[53] Chelbi ST, Wilson ML, Veillard AC, Ingles S, Zhang J (2012). Genetic and epigenetic mechanisms collaborate to control SERPINA3 expression and its association with placental diseases.. Hum Mol Genet.

[54] Morgan K, Kalsheker NA (1997). Regulation of the serine proteinase inhibitor (SERPIN) gene alpha 1-antitrypsin: a paradigm for other SERPINs.. Int J Biochem Cell Biol.

[55] Flach CF, Qadri F, Bhuiyan TR, Alam NH, Jennische E (2007). Broad up-regulation of innate defense factors during acute cholera.. Infect Immun.

[56] O'Connor W, Kamanaka M, Booth CJ, Town T, Nakae S (2009). A protective function for interleukin 17A in T cell-mediated intestinal inflammation.. Nat Immunol.

[57] Sugimoto K, Ogawa A, Mizoguchi E, Shimomura Y, Andoh A (2008). IL-22 ameliorates intestinal inflammation in a mouse model of ulcerative colitis.. J Clin Invest.

[58] Zenewicz LA, Yancopoulos GD, Valenzuela DM, Murphy AJ, Stevens S (2008). Innate and adaptive interleukin-22 protects mice from inflammatory bowel disease.. Immunity.

[59] Glas J, Seiderer J, Bayrle C, Wetzke M, Fries C (2011). The role of osteopontin (OPN/SPP1) haplotypes in the susceptibility to Crohn's disease.. PLoS One.

[60] Delerive P, Monte D, Dubois G, Trottein F, Fruchart-Najib J (2001). The orphan nuclear receptor ROR alpha is a negative regulator of the inflammatory response.. EMBO Rep.

[61] Lees CW, Barrett JC, Parkes M, Satsangi J (2011). New IBD genetics: common pathways with other diseases.. Gut.

[62] van der Heide F, Nolte IM, Kleibeuker JH, Wijmenga C, Dijkstra G (2010). Differences in genetic background between active smokers, passive smokers, and non-smokers with Crohn's disease.. Am J Gastroenterol.

