# Supplementary material for: Analysis of IL12B Gene Variants in Inflammatory Bowel Disease
Source: PLoS One. 2012 Mar 30;7(3):e34349. doi: 10.1371/journal.pone.0034349 (PMC3316707; doi:10.1371/journal.pone.0034349)
Supplement: Table S1 — Primer sequences (F: forward primer, R: reverse Primer), FRET probe sequences, and primer annealing temperatures used for genotyping of IL12B variants. Note: FL: Fluorescein, LC610: LightCycler-Red 610; LC640: LightCycler-Red 640; LC670: LightCycler-Red 670. The polymorphic position within the sensor probe is underlined. A phosphate is linked to the 3′-end of the acceptor probe to prevent elongation by the DNA polymerase in the PCR given based on a median split. (DOC) [file pone.0034349.s001.doc]

**Supplemental Table S1.**

| **Polymorphism** | **Primer sequences** | **FRET probe sequences** |
| --- | --- | --- |
| rs3212227 | F: GAGGAAAAGTGGAAGATATTAAGC  R: AAGGCCCATGGCAACTTG | GCTGTATTTGTATAGTTCGATGCTAA-FL  LC670-GCTGTATTTGTATAGTTCGATGCTAA |
| rs17860508 | F: GGCTGATGCTTGGAGATTGTGAT  R: GTCTGGATTGTGAAGTGGGAC | CTGTCTCCGAGAGAGGGCT-FL  LC640-CCCCCACATTAGAACAACCTGCCAA |
| rs10045431 | F: CAACTTGGCCTGAAGGCA  R: GCACTCCCACGTACCCCAT | AGCCCAGCATTAAACTCTCAAA-FL  LC640-TGATGCCTTGGCCTTGAGACTAGAG |
| rs6887695 | F: CTTCCTGCCTCCGCTAGCC  R: AACACCCCCTAGGTCACAA | TCCAGACTATTGACCACTACACTAC-FL  LC610-CTGCTTCTCTCAAACTGACTTACCGAG |

**Supplemental Table S1.** Primer sequences (F: forward primer, R: reverse Primer), FRET probe sequences, and primer annealing temperatures used for genotyping of *IL12B* variants. Note: FL: Fluorescein, LC610: LightCycler-Red 610; LC640: LightCycler-Red 640; LC670: LightCycler-Red 670. The polymorphic position within the sensor probe is underlined. A phosphate is linked to the 3'-end of the acceptor probe to prevent elongation by the DNA polymerase in the PCR given based on a median split.
