# Supplementary material for: Analysis of IL12B Gene Variants in Inflammatory Bowel Disease
Source: PLoS One. 2012 Mar 30;7(3):e34349. doi: 10.1371/journal.pone.0034349 (PMC3316707; doi:10.1371/journal.pone.0034349)
Supplement: Table S2 — Primer sequences used for the sequence analysis of the IL12B variants. (DOC) [file pone.0034349.s002.doc]

**Supplemental Table S2.**

| **Polymorphism** | **Primer sequences** |
| --- | --- |
| rs3212227 | GAGGAAAAGTGGAAGATATTAAGC  AAGGCCCATGGCAACTTG |
| rs17860508 | GGCTGATGCTTGGAGATTGTGAT  GTCTGGATTGTGAAGTGGGAC |
| rs10045431 | GCTTAGGAGAGCTTGGTCCAT  GGAGGGGCCCCAACCT |
| rs6887695 | CTTCCTGCCTCCGCTAGCC  AACACCCCCTAGGTCACAA |

**Supplemental Table S2.** Primer sequences used for the sequence analysis of the *IL12B* variants.
