# Supplementary material for: Analysis of IL12B Gene Variants in Inflammatory Bowel Disease
Source: PLoS One. 2012 Mar 30;7(3):e34349. doi: 10.1371/journal.pone.0034349 (PMC3316707; doi:10.1371/journal.pone.0034349)
Supplement: Table S3 — Comparison of the association signals of IL12B with the association signals of the three most strongly CD-associated genes NOD2, IL23R and ATG16L1 in the Munich IBD case-control cohort. Minor allele frequencies (MAF), allelic test P-values, and odds ratios (OR, shown for the minor allele) with 95% confidence intervals (CI) are depicted for both the CD and UC case-control cohorts. Details on the analyses of NOD2, IL23R and ATG16L1 gene variants were published in previous studies. (DOC) [file pone.0034349.s003.doc]

**Supplemental Table S3.**

| **SNP** | **Minor allele** | **Crohn’s disease** | | | **Ulcerative colitis** | | | **Controls** |
| --- | --- | --- | --- | --- | --- | --- | --- | --- |
|  |  | **MAF** | **p value** | **OR [95 % CI]** | **MAF** | **p value** | **OR [95 % CI]** | **MAF** |
| *IL12B* rs3212227 | G | 0.209 | 0.684 | 0.97 [0.82-1.13] | 0.214 | 0.777 | 1.03 [0.83-1.29] | 0.209 |
| *IL12B* rs17860508 | TTAGAG | 0.485 | 0.974 | 1.00 [0.88-1.14] | 0.489 | 0.854 | 1.02 [0.85-1.22] | 0.484 |
| *IL12B* rs10045431 | A | 0.271 | 0.258 | 0.92 [0.80-1.06] | 0.252 | *0.083* | 0.83 [0.68-1.02] | 0.288 |
| *IL12B* rs6887695 | C | 0.329 | *0.066* | 1.41 [0.99-1.31] | 0.336 | *0.092* | 1.18 [0.97-1.43] | 0.300 |
| *NOD2*  rs2066843 | T | 0.390 | **3.01 x 10^-5^** | 1.48 [1.23-1.78] | 0.300 | 0.967 | 1.01 [0.85-1.19] | 0.299 |
| *NOD2*  *rs2076756* | G | 0.380 | **4.01 x 10^-6^** | 1.54 [1.28-1.86] | 0.270 | 0.374 | 1.09 [0.94-1.27] | 0.286 |
| *NOD2*  rs2066844 | T | 0.089 | **1.43 x 10^-6^** | 2.07 [1.53-2.79] | 0.046 | 0.749 | 0.92 [0.60-1.40] | 0.050 |
| *NOD2*  rs2066845 | G | 0.042 | **1.10 x 10^-2^** | 1.72 [1.14-2.60] | 0.022 | 1.000 | 0.94 [0.51-1.73] | 0.024 |
| *NOD2*  rs2066847 | insC | 0.121 | **1.88 x 10^-14^** | 5.03 [3.54-7.15] | 0.022 | 0.391 | 0.76 [0.42-1.37] | 0.028 |
| *IL23R* rs1004819 | T | 0.360 | **1.92 x 10^-11^** | 1.56 [1.37-1.78] | 0.314 | **3.81 x 10^-3^** | 1.27 [1.08-1.50] | 0.265 |
| *IL23R* rs7517847 | G | 0.356 | **1.86 x 10^-9^** | 0.68 [0.60-0.77] | 0.381 | **3.78 x 10^-4^** | 0.76 [0.65-0.88] | 0.448 |
| *IL23R* rs11209026 | A | 0.030 | **8.04 x 10^-8^** | 0.43 [0.31-0.59] | 0.049 | **3.61 x 10^-2^** | 0.70 [0.50-0.98] | 0.068 |
| *ATG16L1* rs2241880 | A | 0.410 | **3.70 x 10^-6^** | 0.74 [0.65-0.84] | 0.449 | **7.60 x 10^-2^** | 0.87 [0.74-1.02] | 0.242 |

**Supplemental Table S3.** Comparison of the association signals of *IL12B* with the association signals of the three most strongly CD-associated genes *NOD2, IL23R* and *ATG16L1* in the Munich IBD case-control cohort. Minor allele frequencies (MAF), allelic test *P*-values, and odds ratios (OR, shown for the minor allele) with 95% confidence intervals (CI) are depicted for both the CD and UC case-control cohorts. Details on the analyses of *NOD2, IL23R* and *ATG16L1* gene variants were published in previous studies [1,2,3].

**References**

1. Glas J, Seiderer J, Tillack C, Pfennig S, Beigel F, et al. (2010) The NOD2 single nucleotide polymorphisms rs2066843 and rs2076756 are novel and common Crohn's disease susceptibility gene variants. PLoS One 5: e14466.

2. Glas J, Seiderer J, Wetzke M, Konrad A, Torok HP, et al. (2007) rs1004819 is the main disease-associated IL23R variant in German Crohn's disease patients: combined analysis of IL23R, CARD15, and OCTN1/2 variants. PLoS ONE 2: e819.

3. Glas J, Konrad A, Schmechel S, Dambacher J, Seiderer J, et al. (2008) The ATG16L1 gene variants rs2241879 and rs2241880 (T300A) are strongly associated with susceptibility to Crohn's disease in the German population. Am J Gastroenterol 103: 682-691.
