# Supplementary material for: Analysis of IL12B Gene Variants in Inflammatory Bowel Disease
Source: PLoS One. 2012 Mar 30;7(3):e34349. doi: 10.1371/journal.pone.0034349 (PMC3316707; doi:10.1371/journal.pone.0034349)
Supplement: Table S4 — Overview of published studies on IL12B in patients with inflammatory bowel diseases. CD: Crohn's disease; UC: ulcerative colitis. (DOC) [file pone.0034349.s004.doc]

**Supplemental Table S4.**

| **First author** | **Ethnicity** | ***IL12B* SNP**  **(rs number)** | **Controls (n)** | **CD (n)** | **CD vs. controls**  **(p-value)** | **UC (n)** | **UC vs. controls** | **Reference** |
| --- | --- | --- | --- | --- | --- | --- | --- | --- |
| Anderson | Caucasian (Meta-analysis) | rs6871626 | 15,056 (vs. CD)  19,718 (vs. UC) | 6,333 | P_meta_  6.08 × 10^−12^ | 6,687 | P_meta_  1.02 × 10^−8^ |  |
|  |  | rs6556412 |  |  | P_meta_  5.37 × 10^−14^ |  | P_meta_  1.69 × 10^−5^ |  |
|  |  | rs943072 |  |  |  |  | P_meta_  1.05 × 10^−6^ |  |
| Franke | Caucasian (Meta-analysis) | rs6556412 | 14,026 | 15,694 | 5.37 × 10^−14^ | - | - |  |
| Parkes | European ancestry | rs6887695 | 2,024 | 1,182 | P_rep_=8.4×10^−5^ | - | - |  |
| Peter | Ashkenazi Jews | rs6887695 | 503 | 369 | 0.81 | - | - |  |
|  |  | rs10045431 |  |  | 0.59 |  |  |  |
| Yamazaki | Japan | rs6887695 | 470 | 484 | 0.035 (under the recessive model)  0.12 (allelic) | - | - |  |
| Marquez | Spain | rs6887695 | 547 | 344 | 0.137 | 363 | 0.007 |  |
| Weersma | Netherlands/ Belgium | rs6887695 | 1,086 (vs.CD)  1,045 (vs. UC) | 1,621 | 1.51x 10^-3^ | 1,442 | 3.62 x 10^-2^ |  |
| van der Heide | Netherlands | rs6887695 | 976 | 310 | 0.023 | - | - |  |
| Szperl | Netherlands | rs41292470, (previously  reported as rs17860508) | 1085 | - | - | 940 | 0.025 |  |
|  | Finland |  | 312 |  |  | 345 | 0.06 |  |
|  | combined |  | 1397 |  |  | 1285 | 0.003 |  |
| Barrett | Caucasian (Meta-analysis) | rs10045431 | 4,829 | 3,230 | p-scan  8.80x10^-9^  p-replication 3.66x10^-6^  p-combined  3.86x10^-13^ | - | - |  |
| Ferguson | New Zealand/ Caucasian | rs1363670 | 407 | 339 | 0.338 | - | - |  |
|  |  | rs6887695 |  |  | 0.358 |  |  |  |
| Wang | WTCCC  (European ancestry) | rs1363670 | 1480 | 1748 | 6.89x10^-6^ | - | - |  |
|  | Ped-IBD  (European Ancestry) | rs6556412 | 4250 | 647 | 0.00114 |  |  |  |
|  | CDCC  (European Ancestry) | rs10045431 | 2507 | 1083 | 6.02x10^-7^ |  |  |  |
|  | CHOP-CD-AA  (African Americans) | rs1422878 | 527 | 40 | 0.01253 |  |  |  |
| McGovern | Italy | rs10045431 | 826 | - | - | 993 | 0.024 |  |
|  | Netherlands |  | 754 |  |  | 1016 | 0.023 |  |
|  | Italy, Netherlands |  | combined (826+754=1500 ) |  |  | combined (993+1016=2009) | Combined 1.4x10^-3^ |  |
| Festen | Netherlands | no rs-number mentioned | 1902 | - | - | 1455 | No association shown for *IL12B* |  |
| Törkvist | Sweden | rs10045431 | 1460 | 736 | 0.27 | 935 | 0.20 |  |
| Anderson | UK | rs10045431 | 3028 | 1560 |  | 2527 | 5.21x10^-4^ |  |
| Fisher | UK combined with WTCCC results | rs6556416 (as a proxy for rs10045431) | 1492 + 1470 (WTCCC controls) | - | - | 1740 + 1841 (WTCCC cases) | 6.8x10^-4^ |  |
|  |  | rs6887695 |  |  |  |  | 0.0016 |  |
| Glas | Germany | rs6887695 | 965 | 913 | 0.066 | 318 | 0.092 | Study presented in this manuscript |
|  |  | rs3212227 |  |  | 0.684 |  | 0.777 |  |
|  |  | rs10045431 |  |  | 0.258 |  | 0.083 |  |
|  |  | rs17860508 |  |  | 0.974 |  | 0.854 |  |

**Supplemental Table S4.** Overview of published studies on *IL12B* in patients with inflammatory bowel diseases. CD: Crohn’s disease; UC: ulcerative colitis

References

1. Anderson CA, Boucher G, Lees CW, Franke A, D'Amato M, et al. Meta-analysis identifies 29 additional ulcerative colitis risk loci, increasing the number of confirmed associations to 47. Nat Genet 43: 246-252.

2. Franke A, McGovern DP, Barrett JC, Wang K, Radford-Smith GL, et al. Genome-wide meta-analysis increases to 71 the number of confirmed Crohn's disease susceptibility loci. Nat Genet 42: 1118-1125.

3. Parkes M, Barrett JC, Prescott NJ, Tremelling M, Anderson CA, et al. (2007) Sequence variants in the autophagy gene IRGM and multiple other replicating loci contribute to Crohn's disease susceptibility. Nat Genet 39: 830-832.

4. Peter I, Mitchell AA, Ozelius L, Erazo M, Hu J, et al. (2011) Evaluation of 22 genetic variants with Crohn's disease risk in the Ashkenazi Jewish population: a case-control study. BMC Med Genet 12: 63.

5. Yamazaki K, Takahashi A, Takazoe M, Kubo M, Onouchi Y, et al. (2009) Positive association of genetic variants in the upstream region of NKX2-3 with Crohn's disease in Japanese patients. Gut 58: 228-232.

6. Marquez A, Mendoza JL, Taxonera C, Diaz-Rubio M, De La Concha EG, et al. (2008) IL23R and IL12B polymorphisms in Spanish IBD patients: no evidence of interaction. Inflamm Bowel Dis 14: 1192-1196.

7. Weersma RK, Stokkers PC, Cleynen I, Wolfkamp SC, Henckaerts L, et al. (2009) Confirmation of multiple Crohn's disease susceptibility loci in a large Dutch-Belgian cohort. Am J Gastroenterol 104: 630-638.

8. van der Heide F, Nolte IM, Kleibeuker JH, Wijmenga C, Dijkstra G, et al. (2010) Differences in genetic background between active smokers, passive smokers, and non-smokers with Crohn's disease. Am J Gastroenterol 105: 1165-1172.

9. Szperl A, Saavalainen P, Weersma RK, Lappalainen M, Paavola-Sakki P, et al. (2011) Functional polymorphism in IL12B promoter site is associated with ulcerative colitis. Inflamm Bowel Dis 17: E38-40.

10. Barrett JC, Hansoul S, Nicolae DL, Cho JH, Duerr RH, et al. (2008) Genome-wide association defines more than 30 distinct susceptibility loci for Crohn's disease. Nat Genet 40: 955-962.

11. Ferguson LR, Han DY, Fraser AG, Huebner C, Lam WJ, et al. (2010) IL23R and IL12B SNPs and Haplotypes Strongly Associate with Crohn's Disease Risk in a New Zealand Population. Gastroenterol Res Pract 2010: 539461.

12. Wang K, Zhang H, Kugathasan S, Annese V, Bradfield JP, et al. (2009) Diverse genome-wide association studies associate the IL12/IL23 pathway with Crohn Disease. Am J Hum Genet 84: 399-405.

13. Wellcome Trust Case Control Consortium. (2007) Genome-wide association study of 14,000 cases of seven common diseases and 3,000 shared controls. Nature 447: 661-678.

14. Kugathasan S, Baldassano RN, Bradfield JP, Sleiman PM, Imielinski M, et al. (2008) Loci on 20q13 and 21q22 are associated with pediatric-onset inflammatory bowel disease. Nat Genet 40: 1211-1215.

15. McGovern DP, Gardet A, Torkvist L, Goyette P, Essers J, et al. (2010) Genome-wide association identifies multiple ulcerative colitis susceptibility loci. Nat Genet 42: 332-337.

16. Festen EA, Stokkers PC, van Diemen CC, van Bodegraven AA, Boezen HM, et al. (2010) Genetic analysis in a Dutch study sample identifies more ulcerative colitis susceptibility loci and shows their additive role in disease risk. Am J Gastroenterol 105: 395-402.

17. Torkvist L, Halfvarson J, Ong RT, Lordal M, Sjoqvist U, et al. (2010) Analysis of 39 Crohn's disease risk loci in Swedish inflammatory bowel disease patients. Inflamm Bowel Dis 16: 907-909.

18. Anderson CA, Massey DC, Barrett JC, Prescott NJ, Tremelling M, et al. (2009) Investigation of Crohn's disease risk loci in ulcerative colitis further defines their molecular relationship. Gastroenterology 136: 523-529 e523.

19. Fisher SA, Tremelling M, Anderson CA, Gwilliam R, Bumpstead S, et al. (2008) Genetic determinants of ulcerative colitis include the ECM1 locus and five loci implicated in Crohn's disease. Nat Genet 40: 710-712.
